# Foraging behaviour of the South American sea lion (*Otaria byronia*) in two disparate ecosystems assessed through blubber fatty acid analysis

**DOI:** 10.1038/s41598-020-62178-6

**Published:** 2020-03-31

**Authors:** Alicia I. Guerrero, Guido Pavez, Macarena Santos-Carvallo, Tracey L. Rogers, Maritza Sepúlveda

**Affiliations:** 1 Centro de Investigación y Gestión de Recursos Naturales (CIGREN), Instituto de Biología, Facultad de Ciencias, Universidad de Valparaíso, 2360102 Valparaíso, Chile; 2 Núcleo Milenio INVASAL, 4030000 Concepción, Chile; 3 Departamento de Ciencias Ecológicas, Facultad de Ciencias, Universidad de Chile, 7800003 Santiago, Chile; 4 Evolution and Ecology Research Centre, School of Biological, Earth and Environmental Sciences, University of New South Wales, 2052 Sydney, Australia

**Keywords:** Ecology, Community ecology, Ecosystem ecology, Invasive species

## Abstract

Fatty acids have been widely used as trophic biomarkers in marine mammals. However, for the South American sea lion, the most abundant otariid in the eastern South Pacific, there is no information about blubber fatty acids and their link to diet. Here, we compare fatty acid profiles of sea lions from two distinct oceanographic regions in northern and southern Chile. Their fatty acids vary greatly between regions, suggesting dietary differences at a spatial scale. The fatty acid C22:6ω3 was more abundant in sea lions from the northern region, likely associated with consumption of anchovy, cephalopods, and crustaceans, which are rich in that fatty acid, and have been reported as their main prey items. Sea lions from the southern region were richer in C22:1 and C20:1, characteristic of teleost fish, suggesting a piscivorous diet. Males displayed a more diverse fatty acid composition than females, suggesting a wider trophic niche. Few individual sea lions within the southern region had unusually high levels of C18:2ω6, commonly found in terrestrial environments. This suggests consumption of farmed salmon, whose diet is usually based on terrestrial sources. This demonstrates how human intervention is being reflected in the tissues of a top predator in a natural environment.

## Introduction

Deciphering trophic interactions of top predators is important to understand the structure and functioning of whole ecosystems. The South American sea lion (*Otaria byronia*) is a top predator with a wide distribution range along the eastern Pacific as well as the western Atlantic coasts of South America^1,2^. They are an abundant species, with population estimates of up to 250,000 individuals, and some populations increasing steadily^1,3^. South American sea lions are considered to be opportunistic predators, therefore, their diet is expected to vary in space and time depending on food availability^4^. In fact, decreases in their population in some areas have been linked to declines in the availability of certain prey species^5^. Unsurprisingly Muñoz *et al*.^4^ reported a great variation in diet composition between northern and southern populations of South American sea lions in Chile. Similarly, Sepúlveda *et al*.^6^ demonstrated temporal differences in their diet composition in a population from southern Chile.

Most of our knowledge on the trophic ecology of South American sea lions relies on traditional methods such as stomach and scat content analyses^7,8^, or on observations of feeding events, which are relatively rare and limited in aquatic environments^9–11^. Through these methods, a broad-spectrum diet has been described^12,13^. These methods provide valuable information about recently consumed diets but are not ideal to determine long-term dietary trends, since this would require of repeated sampling throughout long time periods^14,15^. Furthermore, identifying inter-individual variation in diet preferences is very challenging due to, for example, the difficulty of assigning scats to particular individuals in crowded rookeries^16^.

More recently, biochemical methods, such as stable isotopes or fatty acids, have raised as an alternative to decipher dietary patterns over longer time periods^17,18^. Depending on tissue turnover, these biochemical markers (biomarkers)  can provide information on diet consumed over the last hours, weeks, months, or even years^19–27^. Thus, stable isotopes and fatty acids have become particularly useful to infer foraging habits of those animals that are difficult to study *in situ*, such as most marine mammals. The application of stable isotope analysis to South American sea lions in particular, has resulted not only in a better understanding of their general diet in different geographic zones across their distribution^4,15,16^, but also of other aspects of their trophic ecology such as trophic position within the ecosystem^15^, and dietary changes over time^16,28,29^. The analysis of fatty acids is another biochemical method used to study trophic ecology in marine mammals^22,30–32^. The fatty acid profile of an animal is markedly affected by dietary fats^33^. Many of the fatty acids present in the ecosystem are synthesised at the base of the food web by primary producers, and due to the inability of animals to synthesise or modify most of these compounds^34–36^, they are transferred in a conservative manner to higher trophic levels^17^. The relative conservation of fatty acids across trophic levels makes them adequate qualitative biomarkers of trophic interactions^37^. Given the diversity of fatty acids (usually > 20), this technique can theoretically provide better taxonomic resolution (greater power of distinction among prey species) than stable isotope analysis, where usually only two variables are used (e.g. carbon and nitrogen)^38^. In marine mammals, fatty acids are primarily stored in the blubber when food intake exceeds the energy used^39^. Studies on captive animals have proven that blubber fatty acids can successfully track major dietary preferences and shifts in diet overtime^40–43^. This method has been broadly applied to wild animals mostly as a qualitative tool^22,30,32,44,45^, but more recently its quantitative application is attracting increasing scientific attention^46–48^.

Despite the abundance of South American sea lions, to date, our knowledge about their blubber biochemical composition and its dietary implications is almost inexistent. Lutz *et al*.^49^ reported a fatty acid profile for South American sea lions but did not include the animals’ sex, age, or geographical location, nor the number of animals sampled or body site of sample collection. Since this top predator has a wide distribution range, we expect to find differences in blubber fatty acids among populations inhabiting different oceanographic ecosystems.

**Figure 1:**
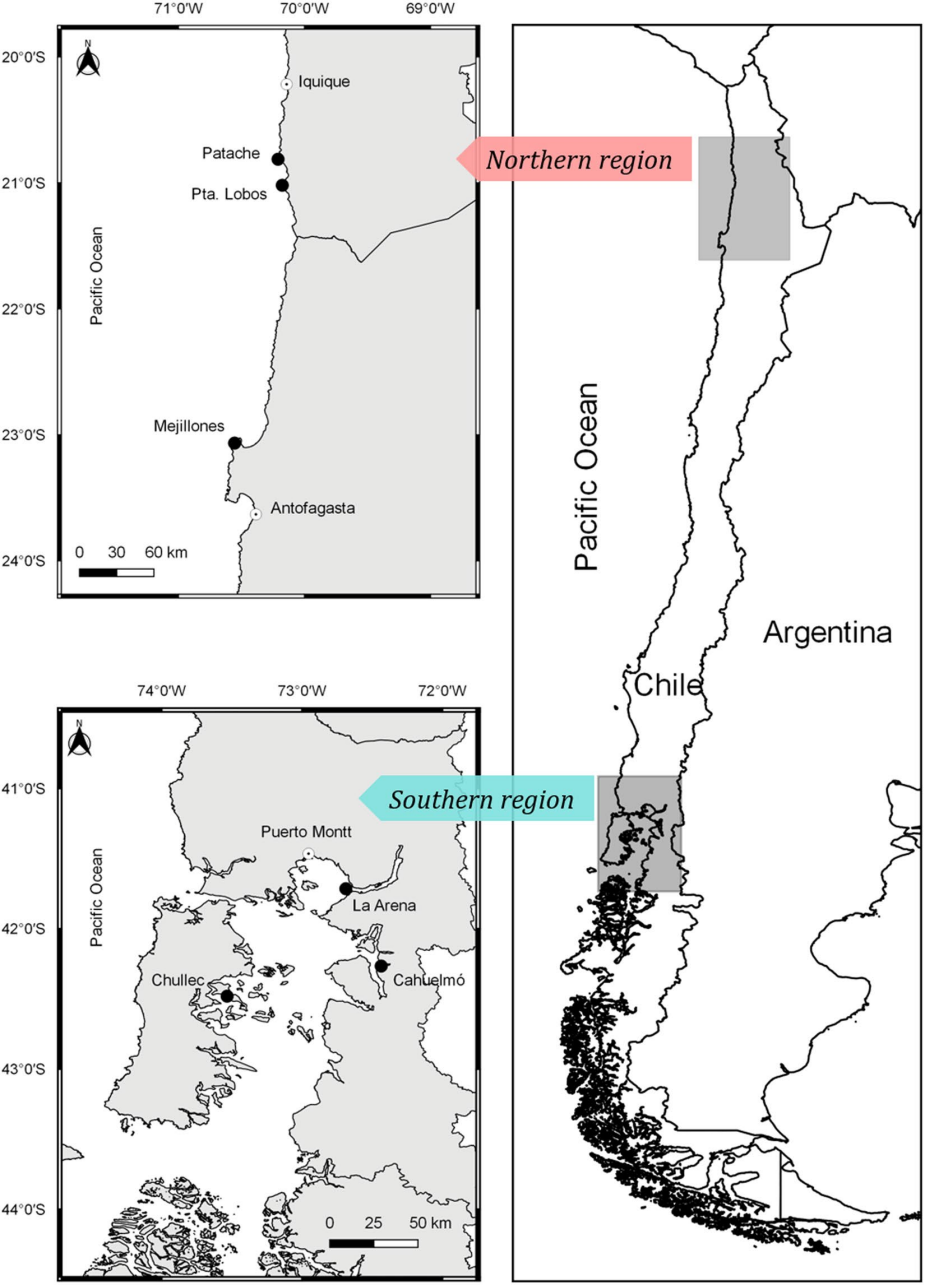
Study sites. Map of the six sampling sites used in this study, three in northern Chile: Patache, Pta. (Punta) Lobos and Mejillones; and three in southern Chile: La Arena, Chullec and Cahuelmó.

The marine ecosystem of the eastern Pacific is highly productive; however marine systems off northern and southern Chile are very different. The northern ecosystem is greatly influenced by the upwelling system of the Humboldt Current; thus, upwelling is active all year round^50,51^. This area has a very narrow, almost inexistent, continental shelf ^52^, resulting in a reduced benthic community and a great abundance of pelagic species. Conversely, the southern Chilean fjord region possesses a wider continental shelf, with the inner sea experiencing seasonal freshwater discharges from ice melt and lakes^53^. In addition, the inner coast has been invaded by salmon farms for about four decades, whose consequences are not yet fully understood. We hypothesize that this will result in dietary differences for populations of South American sea lions from these two geographical locations, which should be reflected in their blubber fatty acids. Although the diet of South American sea lions has been previously studied in these regions via other methods (e.g. stable isotopes^4^ and scat content analyses^54^), we intend to determine whether fatty acids, due to their multivariate nature, can reveal new insights into their foraging behaviour. Thus, the objective of this study was to analyse the blubber fatty acid composition of South American sea lions from two geographical regions supporting completely different ecosystems, in northern and southern Chile (Fig. 1).

Specifically, we intend to: (1) quantify the fatty acid profiles of the blubber of South American sea lions; (2) identify if sea lion fatty acid profiles reflect the spatial variation in diet of sea lions feeding in the two disparate marine ecosystems of northern and southern Chile; and (3) determine whether there are sex differences in fatty acids that would indicate segregation in diet.

## Results

A total of 19 fatty acids were identified consistently in amounts higher than 0.5% in the blubber of South American sea lions (Table 1). Overall, sea lions displayed great individual variation in their blubber fatty acids. The outer blubber layer of sea lions was dominated by monounsaturated fatty acids both in the northern (43.3 ± 10.4%) and the southern region (51.6 ± 14.3%). Saturated fatty acids were the second most abundant (27.7 ± 9.6% and 25.7 ± 6.8% in the northern and southern regions, respectively) followed by polyunsaturated fatty acids which were present in higher proportions in the northern (16.6 ± 12.0%) compared to the southern region (10.5 ± 8.5%). The six most abundant fatty acids, in decreasing order, were: C18:1ω9, C16:0, C16:1ω7, C18:0, C22:6ω3 and C18:1ω7, which accounted for 69.2% and 70.5% of the total fatty acids, in the northern and southern regions, respectively.

**Table 1:** Proportions of fatty acids in the outer blubber layer of the South American sea lion (*Otaria byronia*).

Fatty acid	Northern region						Southern region					
	Total		Females		Males		Total		Females		Males	
	(n = 58)		(n = 37)		(n = 18)		(n = 39)		(n = 19)		(n = 20)	
	Mean	SD	Mean	SD	Mean	SD	Mean	SD	Mean	SD	Mean	SD
**C14:0**	5.1	1.7	5.5	1.6	4.8	1.7	3.0	0.9	3.4	0.7	2.6	0.8
**C15:0**	0.6	0.1	0.6	0.1	0.6	0.2	0.5	0.2	0.4	0.1	0.5	0.3
**C16:0**	14.6	3.7	14.6	3.4	15.3	4.1	13.8	2.7	13.4	2.2	14.1	3.1
**C17:0**	0.5	0.3	0.6	0.2	0.5	0.3	0.5	0.2	0.5	0.2	0.6	0.3
**C18:0**	6.8	3.8	6.1	2.9	8.4	5.0	7.9	4.5	6.1	3.6	9.6	4.7
**∑ SFAs**	**27.7**	**9.6**	**27.3**	**8.3**	**29.5**	**11.4**	**25.7**	**8.5**	**23.8**	**6.8**	**27.4**	**9.1**
**C16:1ω7**	10.0	3.6	10.7	2.9	8.7	4.1	9.0	4.3	11.4	3.9	6.8	3.5
**C17:1**	0.7	0.7	0.7	0.3	0.6	0.3	0.5	0.5	0.5	0.5	0.4	0.5
**C18:1ω9**	25.1	4.2	25.3	4.2	24.6	4.6	31.4	5.9	32.6	4.8	30.2	6.6
**C18:1ω7**	5.4	1.0	5.6	0.9	5.3	1.0	5.4	1.2	5.9	1.2	5.0	1.1
**C20:1ω11**	0.5	0.5	0.5	0.5	0.5	0.5	1.1	0.6	1.3	0.6	0.9	0.6
**C20:1ω9**	1.4	0.5	1.3	0.4	1.4	0.6	3.1	1.4	3.7	1.3	2.6	1.3
**C22:1ω11**	0.2	0.0	0.2	0.0	0.2	0.0	1.0	0.4	1.1	0.4	1.0	0.4
**∑ MUFAs**	**43.3**	**10.4**	**44.2**	**9.1**	**41.2**	**11.2**	**51.6**	**14.3**	**56.7**	**12.6**	**46.7**	**14.0**
**C18:2ω6**	1.2	0.3	1.2	0.3	1.1	0.4	2.0	2.1	1.4	0.6	2.5	2.8
**C18:3ω1**	0.5	1.0	0.4	0.4	0.6	0.6	0.8	0.7	0.6	0.5	1.1	0.9
**C18:4ω3**	0.5	0.3	0.5	0.2	0.6	0.4	0.5	0.2	0.4	0.2	0.6	0.3
**C20:4ω6**	1.5	1.0	1.3	0.9	1.6	1.2	1.3	0.8	0.9	0.5	1.6	0.9
**C20:5ω3**	2.7	1.8	2.9	1.8	2.2	1.8	1.4	0.9	1.4	1.0	1.3	0.8
**C22:5ω3**	3.0	2.2	3.2	2.0	2.5	2.6	1.5	1.1	1.5	1.2	1.5	1.0
**C22:6ω3**	7.2	5.3	7.5	4.9	6.1	6.1	3.0	2.6	3.1	2.7	2.9	2.5
**∑ PUFAs**	**16.6**	**12.0**	**16.9**	**10.6**	**14.6**	**13.0**	**10.5**	**8.5**	**9.3**	**6.7**	**11.6**	**9.1**

SFAs: saturated fatty acids, MUFAs: monounsaturated fatty acids, PUFAs: polyunsaturated fatty acids.

**Figure 2:**
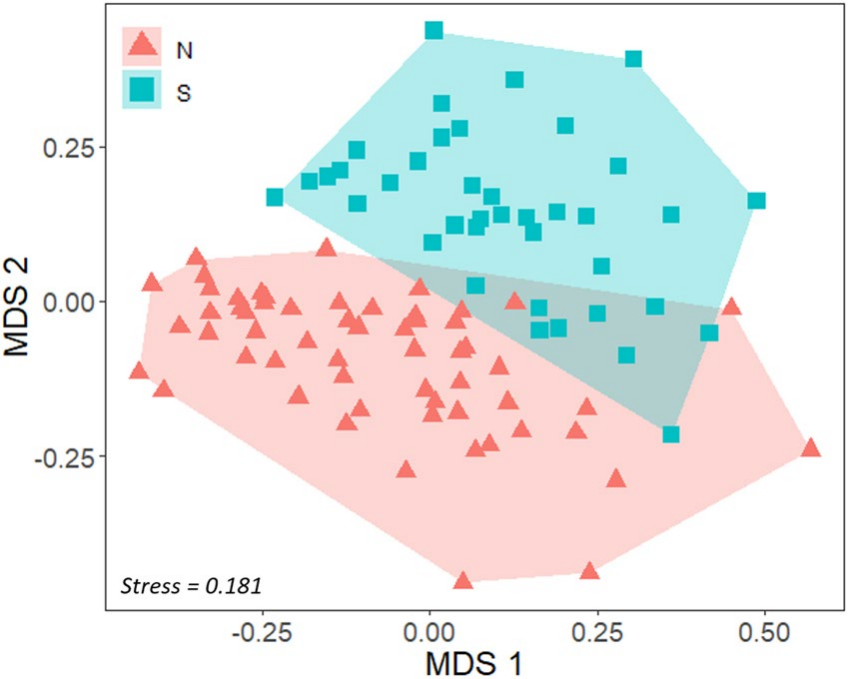
Nonmetric multidimensional scaling (MDS) plot for blubber fatty acids of the South American sea lion (*Otaria byronia*) from the northern (N) and southern (S) region of Chile.

**Figure 3:**
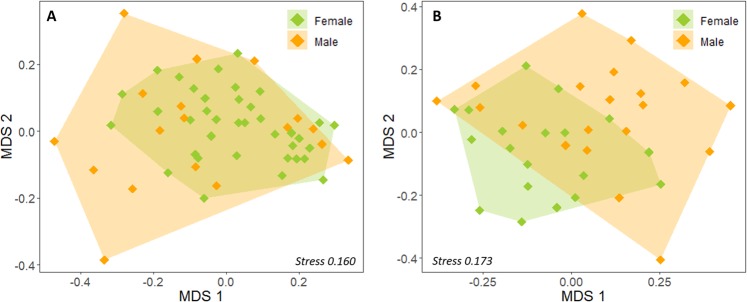
Nonmetric multidimensional scaling (MDS) plot for blubber fatty acids in the South American sea lion within the (**A**) northern and (**B**) southern regions of the study area. Only in the southern region, males and females had statistically different fatty acid compositions (*P* < 0.05).

Both region (PERMANOVA, *F*_1_ = 19.27, *P* = 0.008; Fig. 2) and sex within each region (PERMANOVA, *F*_2_ = 3.37, *P* = 0.008; Fig. 3) influenced the overall fatty acid composition of sea lions. Region explained 17% of the variation whereas sex only explained 6%. SIMPER indicated that the average dissimilarity between northern and southern regions was 24%, and that the fatty acids most responsible for these differences were: C18:1ω9, which was more abundant in sea lions from the southern region, C22:6ω3, which was higher in the northern region, and C18:0, C16:1ω7, C16:0 and C14:0, most of them slightly higher in sea lions from the northern region (Tables 1 and 2). These six fatty acids explained 69% of the variance.

**Table 2:** Contribution of each fatty acid (%) to the dissimilarity between South American sea lions from the northern and southern region, in descending order.

Fatty acid	Contribution to dissimilarity (%)	*P*
C18:1ω9	19.05	**0.001**
C22:6ω3	13.11	**0.027**
C18:0	11.4	0.110
C16:1ω7	11.11	**0.027**
C16:0	8.74	0.952
C14:0	5.66	**0.001**
C22:5ω3	5.25	0.115
C20:1ω9	4.54	**0.001**
C20:5ω3	4.28	0.073
C18:1ω7	2.98	0.117
C18:2ω6	2.52	**0.001**
C20:4ω6	2.25	0.714
C22:1ω11	2.02	**0.001**
C18:3ω1	1.98	0.083
C20:1ω11	1.92	**0.001**
C17:1	1.3	**0.017**

*P* values in bold indicate significant differences between regions.

**Figure 4:**
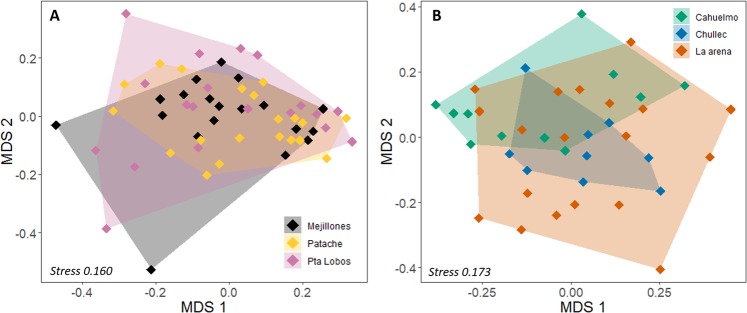
Plot of a nonmetric multidimensional scaling (MDS) analysis of blubber fatty acids of the South American sea lion from three sea lion colonies within (**A**) the northern region, and three colonies within (**B**) the southern region of the study area. Within both regions, blubber fatty acids did not vary by colony (*P* > 0.05).

In multivariate space, males from both regions presented a more widespread distribution compared to females (Fig. 3), whereas sea lion colonies overlapped substantially suggesting similarities in fatty acids (Fig. 4). Within the northern region, blubber fatty acids did not differ significantly by sex (PERMANOVA, *F*_1_ = 2.32, *P* = 0.092; Fig. 3A), sea lion colony (PERMANOVA, *F*_2_ = 0.40, *P* = 0.900: Fig. 4A), or by an interaction of both variables (PERMANOVA, *F*_2_ = 1.11, *P* = 0.363).

Within the southern region, there was a significant difference between sexes (PERMANOVA, *F*_1_ = 6.30, *P* = 0.003; Fig. 3B). SIMPER determined that the average dissimilarity between males and females was 21%, which was mostly driven by higher values of C18:1ω9, C16:1ω7, C22:6ω3, and C20:1ω9 in females, and higher proportions of C18:0, C16:0 and C18:2ω6 in males, accounting for 75% of the variance (Table 3). Conversely, the colony the sea lion belonged to did not influence fatty acid composition (PERMANOVA, *F*_2_ = 1.59, *P* = 0.158, Fig. 4B) and neither did the interaction between colony and sex (PERMANOVA, *F*_2_ = 1.45, *P* = 0.191).

**Table 3:** Contribution of each fatty acid (%) to the dissimilarity between males and females within the southern region, in descending order.

Fatty acid	Contribution to dissimilarity (%)	*P*
C18:1ω9	19.09	0.231
C16:1ω7	15.89	**0.002**
C18:0	15.24	**0.022**
C16:0	8.51	0.569
C22:6ω3	7.04	0.764
C20:1ω9	5.03	**0.004**
C18:2ω6	4.23	0.301
C18:1ω7	4.09	**0.030**
C22:5ω3	3.33	0.625
C14:0	3.15	**0.003**
C20:4ω6	2.67	**0.008**
C20:5ω3	2.58	0.672
C18:3ω1	2.41	0.060
C20:1ω11	2.11	**0.034**
C17:1	1.36	0.327
C22:1ω11	1.11	0.122

*P* values in bold indicate significant differences between sexes.

**Figure 5:**
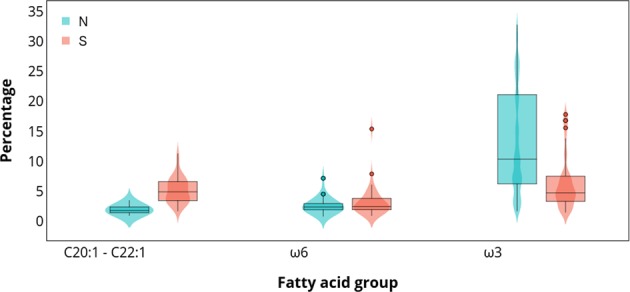
Total percentage of fatty acids with a dietary origin: isomers of C20:1 and C22:1 (C20:1ω9 + ω11 + C22:1ω11 + ω9), ω6 fatty acids (C18:2ω6 + C20:4ω6), and ω3 fatty acids (C18:4ω3 + C20:5ω3 + C22:5ω3 + C22:6ω3), in the outer blubber of South American sea lions from the northern (**N**) and southern (**S**) regions. All these fatty acid groups were statistically different between regions (*P* < 0.05).

The analyses of dietary fatty acid groups revealed that South American sea lions from the northern region have significantly higher proportions of ω3 fatty acids (ANOVA, *F*_1_ = 4.633, *P* = 0.034) but lower proportion of C20:1 and C22:1 isomers (ANOVA, *F*_1_ = 138.4, *P* < 0.001) and ω6 fatty acids (ANOVA, *F*_1_ = 12.46, *P* < 0.001) compared to sea lions from the southern region (Fig. 5).

**Figure 6:**
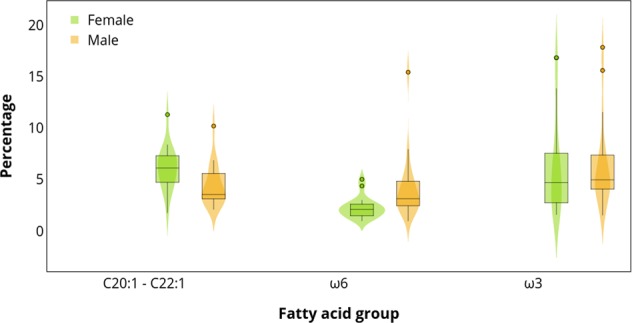
Total percentage of fatty acids with a dietary origin: isomers of C20:1 and C22:1 (C20:1ω9 + ω11 + C22:1ω11 + ω9), ω6 fatty acids (C18:2ω6 + C20:4ω6), and ω3 fatty acids (C18:4ω3 + C20:5ω3 + C22:5ω3 + C22:6ω3), in the outer blubber of South American sea lions from the southern region, separated by sex. Only isomers of C20:1 and C22:1 were statistically different between males and females (*P* < 0.05).

Within the northern region, none of these fatty acid groups were different between males and females (ANOVA, ω3: *F*_1_ = 2.34, *P* = 0.132; isomers: *F*_1_ = 0.075, *P* = 0.786; ω6: *F*_1_ = 0.006, *P* = 0.940). In the southern region, only the proportions of isomers of C20:1 and C22:1 were significantly higher in females (ANOVA, *F*_1_ = 11.3, *P* = 0.002) but ω3 and ω6 fatty acids did not differ statistically between sexes (ANOVA, ω3: *F*_1_ = 0.57, *P* = 0.456; ω6: *F*_1_ = 1.71, *P* = 0.200; Fig. 6).

## Discussion

To our knowledge, this is the first study that describes the fatty acid composition of South American sea lions from different marine ecosystems, including sex classes, and allowing inferences to be made about their dietary patterns. This new approach provides insights into the general foraging behaviour of these sea lions, and not only their short-term diet. This qualitative analysis of fatty acids in a top predator allows us to infer energy flows within the ecosystem, promoting further research based on these trophic biomarkers.

South American sea lions have high proportions of C18:1ω9, C16:0, C16:1ω7 and C18:1ω7, consistent with the main fatty acids found in other marine mammals^32,55,56^. These fatty acids have a mixed origin; they can be synthesised endogenously but are also influenced by diet^46^. Conversely, high amounts of C22:6ω3, or docosahexaenoic acid (DHA), are due to dietary influence, as this type of polyunsaturated fatty acid is synthesised by primary producers^25^. Thus, the abundance of DHA in high trophic levels is associated to consumption of prey rich in DHA and selective retention of this fatty acid by consumers^36^. Although DHA is abundant in the marine environment, its proportion in the blubber of marine mammals depends on diet preferences and hence it can vary greatly among species (e.g., ~1% in dusky dolphins, *Lagenorhynchus obscurus*^57^, and ~13% in short-beaked common dolphins, *Delphinus delphis*^58^), or between populations of the same species (e.g., ~4% versus ~13% in short-beaked common dolphins from North Pacific and North Atlantic oceans, respectively^58,59^). Here, South American sea lions have proportions of DHA that are within the minimum range reported for other otariids^56,60–62^, although with great variability depending on the region (Table 1), as discussed further below.

South American sea lions have high (e.g., ~7%) amounts of C18:0 (Table 1) compared to other carnivorous marine mammals (e.g., where values rarely overcome 3%^30,56,57,61,63,64^). This fatty acid has a mixed origin, where relatively large contributions are from both biosynthesis as well as diet^46^. A high proportion of C18:0 could be a characteristic of the species, where males have higher proportion than females, or may reflect the abundance of this fatty acid in the ecosystem along the eastern South Pacific coast. Only a few studies on fatty acids in the marine ecosystem have been conducted in the area, which include two species of seaweed^65^ and a few species of fish^66^. Seaweeds contain around 8% of C18:0 and fish range between ~6% and ~8%. Thus, since this fatty acid is abundant in organisms of low trophic levels, and due to the little modification of fatty acids passed up the food chain, this top predator should reflect the abundance of this fatty acid in the ecosystem.

As expected, sea lions from the northern region differ in their fatty acid composition compared to those from the southern region as these populations feed within very different oceanographic systems of the eastern South Pacific. Although fatty acid data of potential prey are necessary to establish the diet of a predator in a quantitative manner, the dissimilarities in fatty acids, particularly in those of dietary origin, suggest differences in dietary preferences at this spatial scale. Our findings of this regional dietary difference support previous studies based on stable isotopes that show a great variation in diet composition between northern and southern populations of South American sea lions in Chile^4^. One of the main drivers of segregation was C18:1ω9, which was more abundant in sea lions from the southern region. High proportions of C18:1ω9 are characteristic of many fish species, in particular when C20:1ω9 and C22:1ω11 are also abundant^67^. These other two fatty acids, C20:1ω9 and C22:1ω11, were also identified as responsible of the segregation of sea lions’ fatty acids due to their higher values in the southern region. Copepods synthesise substantial amounts of isomers of C22:1 and C20:1^68^. These fatty acids are conservatively incorporated into the tissues of consumers, in particular fish. Thus, they are characteristic of many teleost fish, along with the fatty acid C18:1ω9^67^, which indicates that the consumption of fish is potentially greater in sea lions from the southern region. This is confirmed by previous studies in the area that report that their diet is dominated by demersal fish^4,6^.

DHA was the second main contributor to dissimilarity between regions, where the proportion of DHA in South American sea lions from the northern region doubled that of sea lions from the southern region. This ω3 fatty acid is a good dietary indicator, which suggests that sea lions from the northern region are feeding on prey rich in DHA, although with great individual variability. Stomach and scat content studies in northern Chile have indicated that the Patagonian squid *Loligo gahi*, the anchovy *Engraulis ringens* and the squat lobster *Pleuroncodes monodon* are the main prey species^54,69^. This coincides with the abundance of DHA in the blubber of sea lions, as cephalopods, crustaceans and fish are rich in DHA, with cephalopods having the highest proportions of DHA within the marine environment^36^. Unlike most fish species, anchovies contain only trace amounts of C22:1 and C20:1 isomers^70^, which explains why sea lions from the northern region, although incorporating fish in their diet, have lower amounts of these isomers compared to sea lions from the south.

The fatty acids C16:ω7 and DHA are also associated to pelagic ecosystems^38^. The abundance of both fatty acids in the northern sea lions suggests that they feed in offshore pelagic habitats. This is consistent with the marine ecosystem off northern Chile where the narrow continental shelf (~200 m)^52^ provides limited opportunity for seafloor benthic foraging. Conversely, sea lions from the southern region inhabit the waters of the inner channel (Fig. 1), which offers more opportunities for benthic feeding. Even if sea lions travelled to more oceanic waters off the outer coast, the continental shelf in this southern area is wider (50 km) than in the northern region^52^; therefore, a more benthic foraging would be expected and has, in fact, been reported by Sepúlveda *et al*.^71^.

Although in the northern region the difference in fatty acid compositions between males and females was not significant, in both regions the pattern is similar: the fatty acids of males are more differentiated from each other, whereas for females the fatty acid composition is more similar (Fig. 3). This would indicate that males have a wider trophic niche than females, and that each male individual have a unique diet that is not very similar to that of its counterparts. Similarly, using stable isotope analysis Sepúlveda *et al*.^6^ showed that subadult and adult males have a broader isotopic niche width than females. This coincides with reports of males travelling longer distances and diving deeper than females^12^, thus, exploring greater areas and increasing the opportunities of having different diets.

Males and females from the northern region did not display differences in their overall fatty acid composition or dietary fatty acids. Potentially, the fact that the study area is larger than that covered in the southern region, implies that there are other sources of variation within the region not accounted for. Further, the number of males and females is unbalanced, which complicates statistical analyses.

In the southern region, sex classes were different in overall fatty acid composition (Fig. 3B) and in proportions of the dietary isomers C20:1 and C22:1 (Fig. 6). According to stomach content studies conducted in the Atlantic coast, males consume a broader trophic spectrum than females^8^. In southern Chile, Sepúlveda *et al*.^6^ found that both males and females only differed slightly in their isotopic values, where both sexes fed mainly on demersal fish, but adult females consumed pelagic fish as their second favourite prey whereas males preferred bentho-pelagic fish. Since fatty acids have greater power of distinction among prey species than stable isotopes^72^; this segregation between sex classes found in this study likely reflects subtle differences in diet. Unfortunately, without fatty acid information of potential prey species, it is difficult to infer whether females are preying more heavily on certain fish species (e.g., richer in C20:1 and C22:1) than males.

Although dietary fatty acids are markedly different between regions, as expected for dissimilar ecosystems, variations among colonies within each region were not evident (Fig. 4). Potentially, individual differences are more important. Using satellite telemetry, Hevia^73^ studied the foraging areas used by South American sea lions from the colony Patache, in northern Chile, and found that sea lions travelled long distances to forage, mainly parallel to the coastline, although a few individuals explored oceanic waters. Other studies on the species have reported segregation in foraging areas for lactating females, where some females are predominantly coastal in their foraging habits whereas others are more pelagic^12^. Similarly, Sepúlveda *et al*.^73^ reported inter-individual differences in δ^13^C values in South American sea lions from southern Chile, suggesting different foraging areas, which matched satellite tracking data derived from the same individuals. This coincides with findings of Muñoz *et al*.^4^, who suggest that South American sea lions display individual trophic specialisation.

High levels of ω6 fatty acids are consistent with terrestrial and freshwater environments, whereas low levels are characteristic of marine ecosystems^68^. For example, when comparing freshwater and marine ringed seals, *Pusa hispida*, Käkelä *et al*.^74^ found that the former had almost five times greater proportion of C20:4ω6 than the marine seal population. Similarly, Smith *et al*.^75^ reported significantly higher proportions of C20:4ω6 and C18:2ω6 in freshwater harbour seals, *Phoca vitulina*, compared to their marine relatives. Other freshwater mammals such as some species of beavers and otters have levels of C18:2ω6 that range from ~10–30%, being sometimes the most abundant fatty acid in their fat depots^76,77^. Here, differences in ω6 values between sea lions from northern and southern regions are important for C18:2ω6 but not for C20:4ω6.

Although sea lions from both regions are marine animals, the southern study area receives an important fresh water discharge, as this area is within one of the most extensive fjord regions in the world^53^. Sea lions from the northern region have levels of C18:2ω6 within the normal range reported for other otariids, which rarely overcomes ~1.5%^56,57,60–62^. Conversely, sea lions from the southern region have higher levels of C18:2ω6 (Table 1, Fig. 5), although with substantial inter-individual variation, where a few sea lions, especially males, had unusually high values of this fatty acid (Fig. 6). C18:2ω6 is characteristic of vascular plants and is considered dietary since their conversion from C18:1ω9 requires Δ12 desaturase enzymes found only in primary producers^78^. This suggests that there is individual specialisation, where most of the population feed on a wholly marine ecosystem, and some sea lions are exploiting prey from inland habitats. South American sea lions are known to enter river mouths and Atlantic coastal lagoons along their distribution zone^79^. In Chile, a very well-known example is a small resident colony that inhabits the Valdivia River^80^, whereas in other areas in southern Chile they have been reported to swim up the rivers to feed on feral salmon^73^. However, sea lions in this study were sampled in a marine area, which indicates that they do not live in the rivers permanently. Thus, an intermittent freshwater foraging would not entirely explain why these sea lion males have values of C18:2ω6 equal or higher than seals that spend their entire lives in freshwater habitats.

Another explanation to higher levels of ω6 fatty acids found in some sea lions in the southern region is the consumption of farmed salmon. This area is characterized by a high concentration of exotic salmon farms. Different studies have reported frequent attacks to these salmon farms by South American sea lions^10,81^ and, through stable isotope analysis, farmed salmon has been identified as one of the main contributors to the diet of South American sea lions, especially for males^4,6,73^. The use of products such as vegetable oil, soybean, poultry and wheat^82–84^ in pellets of farmed salmon contribute to an increase in their proportions of fatty acids of terrestrial origin, such as C18:2ω6. Thus, farmed salmon has been found to contain up to six times higher proportions of ω6 fatty acids compared to wild salmon^83^.

**Figure 7:**
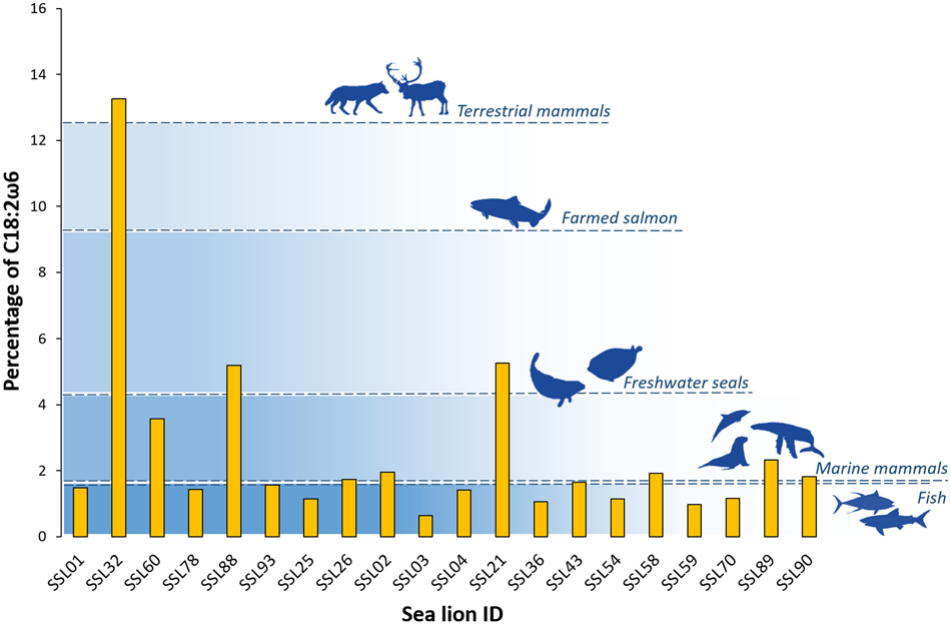
Percentage of C18:2ω6 for individual male South American sea lions from the southern region, some of which have unusually high levels of this fatty acid. These high values likely indicate important intake of farmed salmon by some sea lions. For comparison, horizontal dashed lines indicate average values of different groups of animals. Fish (n = 239), marine mammal (n = 205) and terrestrial mammal (n = 193) values were obtained from Colombo *et al*.^36^. Farmed salmon values (n = 81) correspond to Atlantic salmon, and were calculated from Blanchet *et al*.^83^, Megdal *et al*.^85^, and Aursand *et al*.^86^. Freshwater seal values (n = 34) were calculated from Grahl-Nielsen *et al*.^97^ and Käkelä *et al*.^74^ corresponding to Baikal seals, *Phoca sibirica*, and Saimaa ringed seals, *P. hispida saimensis*, respectively. Images by Alicia I. Guerrero.

Although most male sea lions have C18:2ω6 values within the normal range reported for marine mammals, some of them have C18:2ω6 values comparable to freshwater seals, with one male having values similar to those found in terrestrial mammals (Fig. 7). The figure illustrates the average value of this fatty acid for farmed Atlantic salmon, *Salmo salar*, based on studies conducted in the early 1990s and 2000s^83,85,86^; however, this value is potentially underestimated, as Sprague *et al*.^87^ demonstrates that, between 2006 and 2015, the integration of terrestrial-derived ingredients in the diet of farmed salmon has led to a steady increase of terrestrial fatty acids in farmed Atlantic salmon from Scottish waters. Unfortunately, little is known about the fatty acid composition of farmed salmon in our study area.

Consequently, high levels of ω6 fatty acids in the blubber of sea lions are potentially related to heavy consumption of farmed salmon by a few individuals, since freshwater foraging would not explain values as high as those of terrestrial mammals. The consumption of farmed salmon is a foraging strategy adopted by only a part of the population^73^. For example, Muñoz *et al*.^4^, using stable isotope analysis, found that whereas salmon contributed more than 50% to the diet of a few South American sea lions, for others the contribution of salmon was insignificant. Similarly, using satellite tracking, Sepúlveda *et al*.^73^ determined that of 8 sea lions tracked, only one spent more than 20% of the time within close proximity (1 km) of salmon farms whereas the other 7 spent between 0.1 and 8.5%. Additionally, the median contribution of farmed salmon to the diet of these 8 sea lions, estimated through hair stable isotopes, ranged from 8 to 48%^73^.

Here, all values of C18:2ω6 higher than 4% correspond to adult and subadult males. Coincidently, salmon consumption is a foraging strategy adopted mainly by subadult and adult males, the age/sex classes most frequently involved in interactions with salmon farms in the region^81^. This is the first study, to our knowledge, to demonstrate how human-derived food has introduced a foreign fatty acid into the tissues of a top predator living in a natural marine ecosystem. Whether these unusual fatty acid signatures are also present in organisms at lower trophic levels, is something that remains to be investigated.

Overall, the dietary inferences that we made based on blubber fatty acids of the South American sea lion concord with our current knowledge about its foraging habits; however, more accurate, and quantitative, results could be obtained if fatty acid data of potential prey species were available. In order to understand trophic interactions, further studies on fatty acid composition of different marine organisms including farmed and feral salmonids are needed, in particular in the eastern South Pacific.

## Conclusions

This study demonstrated that sea lions from different oceanographic regions have contrasting blubber fatty acids, which reflects variations in prey availability. Sea lions from the northern region prefer prey rich in C22:6ω3, like anchovy, cephalopods and crustaceans, whereas southern sea lions consume more fish, rich in C22:1ω11, C20:1ω9 and C18:1 ω9. Unusually high levels of C18:2ω6, a fatty acid abundant in terrestrial environments, in sea lions from the southern region would indicate consumption of farmed salmon and demonstrates how salmon farming is potentially introducing a foreign compound into a natural ecosystem.

## Materials and Methods

### Sample collection

South American sea lions (n = 97) were sampled in two main areas in the north and south of Chile, which from here on will be referred to as the northern and southern regions, respectively. The three northern study sites, located between 20°48’S and 23°06’S, were Patache, Punta (Pta) Lobos, and Mejillones. More than 2,000 km southwards, between 41°42’S and 42°28’S, the three southern study sites, La Arena, Chullec and Cahuelmó, are sea lion colonies within the inner sea of the Los Lagos Region of southern Chile (Fig. 1). This area is characterised by a high number of salmon farming installations. Field work was conducted during the austral autumn of 2015 (Table 4).

**Table 4:** Number of South American sea lions per age/sex class, sampled at each sea lion colony within the northern and southern regions.

Colony	Northern region			Southern region			*Totals*
	Patache	Pta Lobos	Mejillones	La Arena	Cahuelmó	Chullec	
Subadult males	1	2	3	7	5	2	***20***
Adult males	0	12	0	5	3	0	***20***
Adult females	17	5	15	7	3	7	***54***
Unidentified	2	0	1	0	0	0	***3***
***Totals***	***20***	***19***	***19***	***19***	***11***	***9***	***97***

All applicable national guidelines for the care and use of animals were followed, and the study was approved by the Institutional Committee of Bioethics for Animal Research (CIBICA) of the University of Valparaíso, Chile. Samples were remotely obtained using a hollow-tipped biopsy dart fired from a PaxArms .22 rifle that extracts a small sample of outer blubber, skin, and hair, with minimal disturbance of the animal. Date, sample site, and age/sex class of the individual were registered for each sampling event. Three age/sex classes were considered: (1) subadult males, (2) adult males, and (3) adult females, according to differences in size, body shape, coloration and, in the case of adult males, the presence of a developed mane^2^. Juveniles were not included in our study due to their small sample size. For this study we only used the outer blubber. Although the inner layer of the blubber (the section closest to the muscle) is known to be actively involved in the dietary metabolism reflecting the last weeks to months of feeding^88,89^, here, due to the sampling method utilised, we use only the outer layer (the section just beneath the skin), which has a more structural role^90^, is more stable and thought to reflect a longer-term diet than the inner layer (more than 2–3 months)^35,91^. Thus, dietary inferences made here correspond to a general diet, rather than represent a specific time period.

### Laboratory procedures

Lipid extraction was conducted following a modified Folch *et al*.^92^ method described in Budge *et al*.^35^. In brief, samples were left overnight in a solution containing 2:1 chloroform: methanol and 0.01% of butylated hydroxytoluene. Samples were then homogenised, washed in a salt solution, centrifuged to separate components, dried over anhydrous sodium sulphate and evaporated using nitrogen. Lipids obtained from the procedure were weighted and used for fatty acid transesterification.

We used an acidic transesterification method^35^, using Hilditch reagent, to prepare fatty acid methyl esters (FAMEs), which were then extracted into hexane and stored in vial tubes for gas chromatography analysis.

Gas chromatography analyses of FAMEs were conducted at the Mark Wainwright Analytical Centre within the University of New South Wales (Sydney, Australia). FAMEs were analysed in an Agilent 7890 A Series Gas Chromatograph System (Agilent Technologies, U.S.A.) equipped with a flame ionization detector, and using a flexible fused silica column DB-23 of 30 m length × 0.25 mm ID × 0.25 µm film (Agilent Technologies, U.S.A.). Operating parameters of the gas chromatograph have been described in Guerrero and Rogers^32^. FAMEs were identified using a range of standard mixtures (Nu Check Prep., Elysian, MN, USA) and concentrations were converted to percentage contributions of the total fatty acids.

### Data analysis

Due to noise contribution of fatty acids found in trace amounts^93^, only those fatty acids found in proportions higher than 0.5% were used for statistical analyses. Subadult and adult males were combined into a single group, due to their small sample sizes, making comparisons more numerically balanced. In order to assess separation of groups in multivariate space, we used nonmetric multidimensional scaling (MDS) analysis on untransformed fatty acid data. Permutational multivariate analysis of variance (PERMANOVA) based on Bray-Curtis dissimilarities was used to determine differences in fatty acid composition between groups. In order to account for the effect of sex on the differences in fatty acids between regions, we used a nested PERMANOVA, where sex was nested within each region. Using a two-way PERMANOVA, we tested for differences among colonies and sexes separately for each region, including sex, colony and an interaction term as independent variables. Fatty acids primarily influencing the separation among groups were identified using the similarity percentages routine (SIMPER). All these analyses were conducted using the R package “vegan”^94^.

Additionally, we tested for differences in dietary fatty acids typically used as trophic biomarkers, which included: total proportion of ω3 and ω6 fatty acids, and total proportion of isomers of C20:1 and C22:1. Prior to these statistical analyses, we applied the log-ratio transformation introduced by Aitchison^95^ to our data. Although Budge *et al*.^35^ recommended the use of C18:0 as the reference fatty acid for this transformation, here this compound varied substantially between sexes and regions (Table 1); therefore, we used C15:0 instead, since this fatty acid provides little dietary information, was reliably quantified, and did not contribute to differences between regions and/or sexes. Thus, we used Analysis of Variance (ANOVA) to evaluate differences in these fatty acid groups between regions, and sex within each region. The level of significance for all statistical tests was set at *P* < 0.05. All analyses were conducted using R Studio^96^.

## Data Availability

All data supporting the conclusions of this article are within the paper.
